# A Review on Enterocin DD14, the Leaderless Two-Peptide Bacteriocin with Multiple Biological Functions and Unusual Transport Pathway

**DOI:** 10.3390/antibiotics12071188

**Published:** 2023-07-14

**Authors:** Rabia Ladjouzi, Elodie Dussert, Radja Teiar, Yanath Belguesmia, Djamel Drider

**Affiliations:** UMR Transfrontalière BioEcoAgro, INRAe 1158, ICV—Institut Charles Viollette, University Lille, INRAE, University Liège, UPJV, YNCREA, University Artois, University Littoral Côte d’Opale, F-59000 Lille, France; rabia.ladjouzi@univ-lille.fr (R.L.); elodie.dussert@univ-lille.fr (E.D.); radja.teiar@polytech-lille.fr (R.T.)

**Keywords:** Enterocin DD14 (EntDD14), leaderless bacteriocin (LLB), specific transport, antibacterial and antiviral properties

## Abstract

Enterocin DD14 (EntDD14) is a two-peptide leaderless bacteriocin (LLB) produced by *Enterococcus faecalis* 14, a human strain isolated from meconium. Studies performed on EntDD14 enabled it to show its activity against Gram-positive bacteria such as *Listeria monocytogenes*, *Clostridium perfringens*, *Enterococcus faecalis*, and *Staphylococcus aureus*. EntDD14 was also shown to potentiate the activity of different antibiotics such as erythromycin, kanamycin, and methicillin when assessed against methicillin-resistant *Staphylococcus aureus* (MRSA) in vitro and in vivo in the NMRI-F holoxenic mouse model. Additionally, EntDD14 has an antiviral activity and decreased the secretion of pro-inflammatory IL-6 and IL-8 in inflamed human intestinal Caco-2 cells. The genome of *E. faecalis* 14 was sequenced and annotated. Molecular tools such as Bagel4 software enabled us to locate a 6.7kb-EntDD14 cluster. Transport of EntDD14 outside of the cytoplasm was shown to be performed synergistically by a channel composed of two pleckstrin-homology-domain-containing proteins, namely DdE/DdF and the ABC transporter DdGHIJ. This latter could also protect the bacteriocinogenic strain against extracellular EntDD14. Here, we focus on academic data and potential therapeutic issues of EntDD14, as a model of two-peptide LLB.

## 1. Introduction

Bacteriocins are ribosomally synthesized antimicrobial peptides (AMPs), which are produced by virtually all bacteria [1]. These potent AMPs can kill or inhibit bacterial strains closely related or non-related to the bacteriocinogenic strain (strain producing bacteriocin), but will not harm the producer because of protection ensured by the so-called self-immunity systems [2,3]. The activity towards closely related strains allows ecological and nutritional advantages for the bacteriocinogenic strain. Bacteriocins can act on the target cell, either by disrupting the cell membrane, inducing pore formation, and therefore dissipation of cell contents (metabolites and ions), inhibiting peptidoglycan synthesis, interfering with DNA transcription and replication mechanisms, or preventing protein synthesis by interfering with ribosome function [4,5,6,7]. In the context of a well-acknowledged antibiotic crisis, there are societal expectations and governmental instructions to develop novel strategies to fight bacterial infections. Indeed, the number of deaths directly caused by antibacterial resistance is about 1.27 million per year worldwide, according to a recent study [8], a number expected to increase in the near future, if actions are not taken. A panel of strategies is steadily recommended and includes the use of antimicrobial peptides, such as bacteriocins, which warrant serious consideration [9]. This review focuses on enterocin DD14 (EntDD14), as a model of leaderless bacteriocin (LLB), produced by *Enterococcus faecalis* 14, a strain isolated from meconium [10]. EntDD14, which is a two-peptide (EntDD14A and EntDD14B) LLB, is largely studied and was proven to be a good candidate for therapeutic applications, not only as an antibacterial agent but also as an antiviral agent.

## 2. Diversity and Structures of Leaderless Bacteriocins

**Figure 1:**
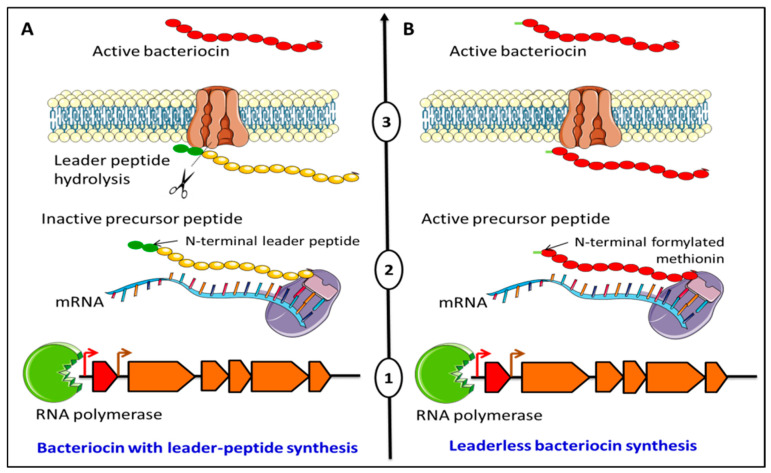
Schematic model for bacteriocin biosynthesis with (**A**) and without (**B**) leader peptide. Bacteriocins with leader peptides (**A**) are transcribed and then translated into an inactive pre-peptide. During export across the plasma membrane, the leader peptide is energetically hydrolyzed, leading to the active form of the precursor. In the case of leaderless bacteriocins, they are transcribed and then translated into an active peptide, often with an N-terminal formylated methionine, which is exported across the membrane.

Depending on the presence or absence of a sequence coding for a leader peptide in the genetic sequence of bacteriocins, two categories of bacteriocins are produced (Figure 1). The first is synthesized as a pre-peptide with an N-terminal leader peptide. This leader peptide is then hydrolyzed when the bacteriocin is exported across the cell membrane resulting in the functional molecule (Figure 1A). The leader peptide was established to play a role in lowering the antimicrobial activity by the unprocessed bacteriocin while it is inside the producer cell and in recognition by the transport machinery that is involved in the translocation of the bacteriocin across the membrane, by ensuring a suitable conformation, which is essential for peptide–target recognition [11,12,13]. The second category of bacteriocins is synthesized without N-terminal extension, and these bacteriocins are hence named leaderless-bacteriocins (LLBs). LLBs lack a leader peptide, do not undergo post-translational modification or processing, and are thought to be active immediately after their translation in the cytoplasm (Figure 1B). Therefore, understanding how LLBs are recognized by dedicated transporters and how the bacteriocinogenic strains protect themselves from the cytotoxic effect of their own LLB need to be elucidated.

Recent studies have shown that LLBs are composed of one to six peptides of a size ranging from 27 to 70 amino acids (Table 1). These studies also underlined high pI (isoelectric-point) values, net cationic charges, significant sequence homologies with most members of LLB subgroups, and an N-terminal formylated methionine. The presence of a formylated methionine at the N-terminal region was reported for LLBs such as bacin [14], thucin [15], toyonsin [16], and enterocin 7 [17]. The removal of the N-terminal formylated methionine, upon cyanogen bromide treatment, had no effect on the bioactivity of lacticin Q or auriocin 53 [18]. In this light, we chemically synthesized EntDD14A and EntDD14B, two peptides constituting EntDD14, with a non-formylated methionine at their N-terminal regions, and the resulting EntDD14 was active against Gram-positive bacteria (data to be published elsewhere). Heterologous expression of LLBs such as aureocin A53 and/or lacticin Q producing N-terminal unformylated methionine peptides were able to demonstrate intact antimicrobial activity [18]. Taken together, these data suggest that N-terminal formylated methionine has apparently no direct role in the antibacterial properties of LLBs.

**Figure 2:**
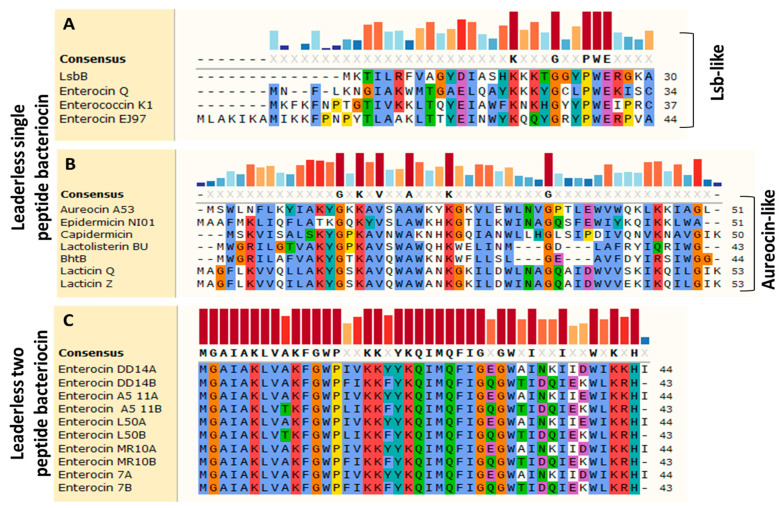
Clustal omega alignments of the Lsb-like (**A**), Aureocin-like (**B**) and the leaderless-two peptide bacteriocins (**C**). The consensus sequences are shown in bold and colored bars. The amino-acid residues are highlighted by Clustal X based on their properties and conservation.

The alignment of the peptide sequences of single-peptide LLBs enabled distinguishing between the Lsb-like group and the aureocin-like group. Consequently, weissellicins Y and M and toyoncin are strikingly different and therefore do not match these two groups. Thus, the Lsb-like group was represented by LsbB, EntQ, EntK1, and EntEJ97. This group has a non-conserved N-terminal region and a very similar C-terminal region with conserved sequences containing Lys17, Gly21, Pro24, Trp25, and Glu26 residues (Figure 2A). The aureocin-like group includes aureocin A53, epidermicin NI01, capidermicin, lactolisteins BU and BHT-B, and lacticins Q and Z. These bacteriocins exhibited conserved sequences with Gly14, Lys16, Val18, Lys25, and Gly35 residues, in reference to the structure of aureocin A53 (Figure 2B). It should be noted that the aureocin-like group is rich in Trp residues, which facilitate their interaction with bacterial membranes [19]. Two-peptide LLBs include enterocin L50 (L50A and B), enterocin MR10 (MR10A and B), enterocin DD14 (EntDD14A and EntDD14B), Ent A5-11 (A5-11-A and B) and Ent 7 (Ent 7A and B) (Table 1). These bacteriocins produced by different strains of *Enterococcus* are highly similar if not identical (Figure 2C). Following this, multi-peptide LLBs include those composed of three, four, or six peptides. In the case of thuricin, a six-peptide LLB, genes thnA5 and thnA6 encode for an identical peptide (Table 1).

**Table 1:** Leaderless group bacteriocins and their relevant properties.

	Bacteriocin	Length (AA)	MW (Da)	pI	Net Charge	Hydrophobicity	Producer Bacterium	Reference
**Single peptide**	Enterocin Q	34	3979.73	9.78	4	−0.359	*E. faecium* L50	[20]
	Aureocin A53	51	6012.19	10.73	8	−0.08	*Staphylococcus aureus A53*	[21]
	LsbB	30	3437.99	10.75	6	−0.683	*Lactococcus lactis* subsp. *lactis* BGMN1-5	[22]
	Enterocin EJ97	44	5350.29	10.39	6	−0.589	*E. faecalis* EJ97	[23]
	BHT-B	44	5193.06	10.57	4	0.241	*S. rattus* strain BHT	[24]
	Lacticin Q	53	5926.06	10.84	6	0.3	*L. lactis* QU 5	[25]
	Lacticin Z	53	5968.1	10.63	5	0.28	*L. lactis* QU 14	[26]
	Weisselicin Y	42	4923.68	10.36	5	−0.09	*Weissella hellenica* QU 13	[27]
	Weissellicin M	43	4966.85	10.39	5	0.037	*W. hellenica* QU 13	[27]
	Epidermicin NI01	51	6072.27	10.95	9	−0.02	*S. epidermidis* strain 224	[28]
	BacSp222	50	5921.92	10.09	4	−0.304	*S. pseudintermedius*	[29]
	Enterocin K1	37	4592.37	10.24	6	−0.7	*E. faecium* EnGen0026	[30]
	Lactolisterin BU	43	5163.02	10.72	5	−0.151	*L. lactis* subsp. *lactis. diacetylactis* BGBU1-4	[31]
	Capidermicin	50	5464	10.22	5	−0.064	*S. capitis* CIT060	[32]
	Toyoncin	70	7817.1	9.94	4	−0.02	*Bacillus toyonensis* XIN-YC13	
**Two peptides**	Enterocin DD14 (MR10) (Ent7)						*E. faecalis* MRR 10-3; 14; 710C	
	DD14A (MR10A) (Ent7A)	44	5204.32	10.68	7	0.202		[33,34,35]
	DD14B (MR10B) (Ent7B)	43	5210.24	10.95	7	−0.109		
	Enterocin L50						*E. faecium* L50	[36]
	L50A	44	5218.35	10.68	7	0.202		
	L50B	43	5206.25	10.95	7	−0.144		
	Enterocin A5-11						*E. durans*	[37]
	A5-11A	44	5190.31	10.57	7	0.202		
	A5-11B	43	5178.22	10.83	7	−0.144		
**Three peptides**	Garvicin KS						*L. garvieae* KS1546	[30]
	KS-A	34	3481.14	10.98	4	0.403		
	KS-B	34	3188.84	11.02	5	0.691		
	KS-C	32	3127.76	10.98	4	0.588		
	Cereucin X						*B. cereus* BAG2O-1	[30]
	X-A	27	2972.48	10.33	2	0.004		
	X-B	29	3165.63	9.92	3	−0.186		
	X-C	30	2826.25	10.64	4	0.52		
	Cereucin V						*B. cereus* VD148	[38]
	V-A	30	3142.69	11.08	3	0.373		
	V-B	30	2857.41	10.87	4	0.65		
	V-C	31	3004.52	10.88	3	0.69		
	Thucin						*B. thuringiensis* P86	[15]
	ThuA1	48	5552.02	10.8	3	0.302		
	ThuA2	48	5578.07	10	3	0.202		
	ThuA3	48	5609.06	10.8	3	0.194		
**Four peptides**	Aureocin A70						*S. aureus* A70	[39]
	A70A	31	2952.53	10.98	4	0.529		
	A70B	30	2825.34	10.87	4	0.707		
	A70C	31	2982.56	10.87	5	0.552		
	A70D	31	3114.76	10.7	4	0.632		
	Cereucin H						*B. cereus* HuA2-4	[38]
	H-A	26	2876.39	10.03	2	0.154		
	H-B	30	3170.7	10.5	2	0.233		
	H-C	30	2867.42	10.87	4	0.667		
	H-D	30	3018.63	10.86	4	0.62		
	Bacin						*Bacillus* sp. TL12	[14]
	BacA1	48	5637.08	10.78	3	0.24375		
	BacA2	48	5652.06	10.26	3	0.1479		
	BacA3	48	5591.09	10.78	3	0.2479		
	BacA4	48	5577.08	10.78	3	0.2416		
**Six peptides**	Thuricin						*B. thuringiensis* LX43	[40]
	Thn A1	48	5396.98	9.82	2	0.308		
	Thn A2	48	5382.96	10.27	2	0.404		
	Thn A3	48	5322.99	9.99	2	0.45625		
	Thn A4	48	5350	9.99	2	0.4		
	Thn A5	48	5364.02	9.99	2	0.40625		
	Thn A6	48	5364.02	9.99	2	0.40625		

**Figure 3:**
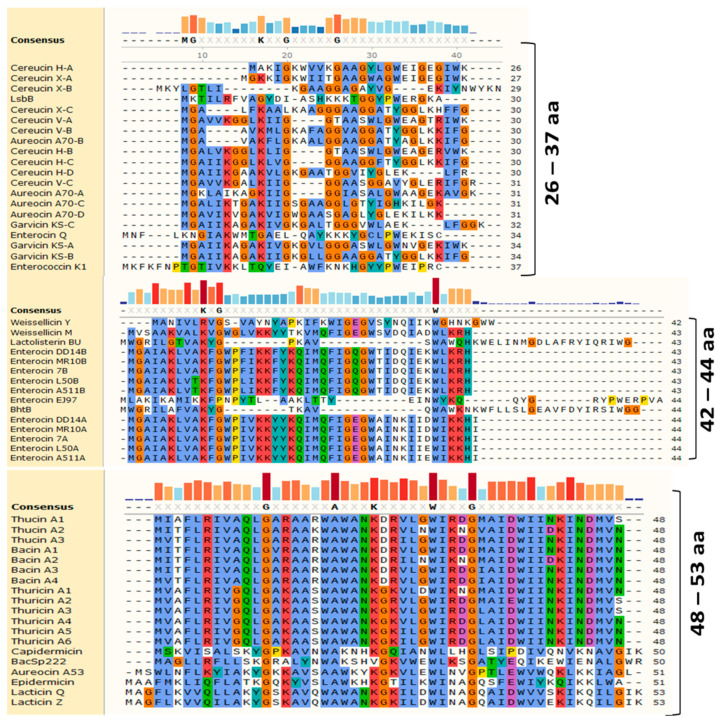
Clustal omega alignments of the leaderless peptide bacteriocins. The consensus sequences are shown in bold and colored bars. The amino-acid residues are highlighted by Clustal X based on their properties and conservation.

The alignment of LLBs based on the number of amino acid residues (aa) of which they are composed enabled us to distinguish three subgroups (Figure 3). The first subgroup comprises bacteriocins with 26 to 37 aa, the second subgroup consists of bacteriocins with 42 to 44 aa, and the third subgroup covers bacteriocins with 48 to 53aa. Toyoncin is excluded from these subgroups as it has 70 aa and has no significant homology with other bacteriocins.

**Figure 4:**
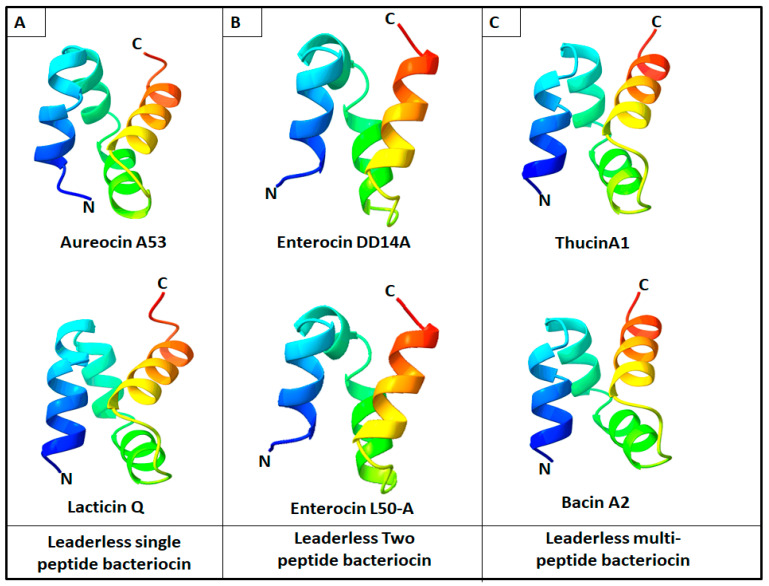
Representations of some examples of leaderless bacteriocin obtained by AlphaFold 2 and visualized by ChimeraX. (**A**) Leaderless single peptide bacteriocin aureocin A53 and lacticin Q; (**B**) Leaderless two peptide bacteriocin EntDD14A and enterocin L50-A; (**C**) Leaderless multi-peptide bacteriocin thucin A1 and bacin A2. N-terminal and C-terminal regions are mentioned as N and C, respectively. Each helix is colored a different color starting with the N-terminus, blue; α-helix 1, light blue; α-helix 2, green; α-helix 3 and orange; α-helix 4.

The small bacteriocins (26–37 aa) displayed structural diversities, with some residues significantly conserved, such as Met1, Gly2, Lys10, Gly13, and Gly15, in reference to the aureocin 70 sequence. The subgroup of bacteriocins 42 to 44 aa appeared to be more homogeneous, with an N-terminal region conserved containing Lys10, Gly12, and Trp39 in reference to the sequence of EntDD14. Finally, the subgroup of bacteriocins of 48–53 aa resulted to be highly homogeneous, with conserved residues such as Gly14, Lys/Arg16, Ala17, Trp20, Ala21, Trp22, Lys25, Leu29, Trp31, and Gly35 and Trp40 and Leu/Ile45 in reference to the sequence of aureocin A53A. These alignments enabled the identification of common residues that could be involved in the antibacterial activity and biosynthetic pathways of LLB. Analysis of structures of some groups of bacteriocins, particularly LLB, and most of circular bacteriocins (Figure 4) revealed the presence of a structural saposin-like fold motif or a related α-helical bundle (Figure 4). The saposin fold referred to the compact structural confirmation forming a small hydrophobic core by four or five amphipathic α-helices [41,42,43]. Notably, the saposin-like fold or an α-helical bundle contains no cysteines, unlike saposins and SAPLIPs, which contain conserved cysteines involved in the formation of disulfides between helices [44]. Common LLB functional properties may be due in part to the fact that they share these similar structural motifs, as the amphipathic helices packing hydrophobic residues produces a hydrophobic core. Bacteriocins are known to interact with bacterial membranes, and the presence of this structural motif can play a major role in interactions with lipids [44,45].

## 3. The Synthesis and Genetic Organization of the Leaderless Bacteriocins

**Figure 5:**
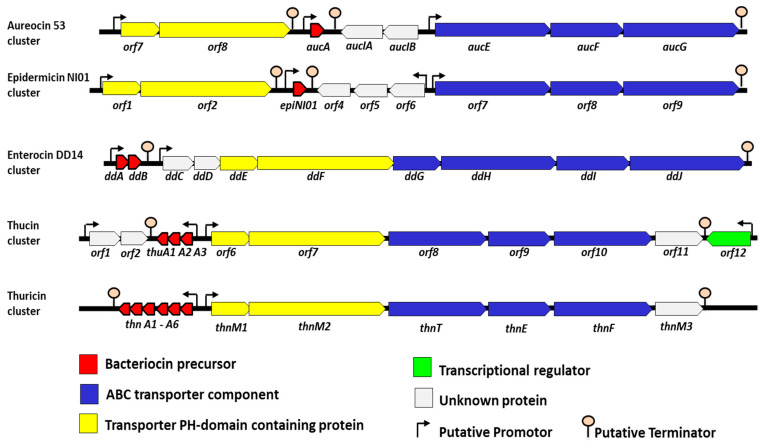
Genetic organization of the biosynthetic gene cluster of leaderless bacteriocins. Genes are represented by a colored arrow coded based on their known and putative functions as indicated. The putative promoters and putative terminators are also represented. Genes are drawn without scale.

The genetic region responsible for LLB synthesis consists of a group of genes organized into one or more operons. These genes include structural genes, which encode precursor peptides, genes encoding transport and/or immune proteins, and sometimes regulatory genes. Clusters of some representative LLBs are depicted in Figure 5. Next, studies performed on aureocin A53 [46] lacticin Q or lacticin Z [47,48,49], aureocin A70 [39,50], and EntDD14 [51,52,53] permitted to gain insights into their genetic organization and genes associated functions. Nevertheless, the mechanisms involved in their immunity and transport outside the bacteriocinogenic strains remain to be understood. The genetic determinants of LLB are either plasmid-based, as in the case of EntL50 [36], and EntQ [20,54], or chromosomal-based, as in the case of weissellicins Y and M [27].

In terms of regulation, some studies reported the role of transcriptional regulators. In the case of aureocin A70 [55], a complex mechanism involving AurR (a helix-turn-helix), and a phage regulator φ11 was reported to regulate this LLB [55]. Next, the LnqR protein (member of the TetR family of transcriptional regulators) was reported to regulate positively the gene transcription and thus, the synthesis of lacticin Q [49]. Moreover, environmental factors such as nutrition-adaptation or temperature variation were reported to control the biosynthesis of weissellicins produced by *Weissella hellenica* QU 13 [27], and EntL50 and EntQ produced by *E. faecium* L50 [54]. In the case of EntDD14, the comparative transcriptomic study conducted on *E. faecalis* 14 and its *Δbac* mutant, lacking the genes coding for EntDD14 revealed the enterocin EntDD14-associated function. These functions include biofilm-formation capability, metabolic reprogramming, and resistance to environmental stresses [56]. For EntDD14 synthesis, we recently established that mRNA stability plays a role in its synthesis via transcription-translation efficacy controlled by the well-known 3′-5′ exoribonuclease, polynucleotide phosphorylase PNPase (submitted).

It was established that bacteriocin synthesis was generally correlated with the expression of specific immunity proteins, allowing for the protection of the bacteriocinogenic strain from the toxicity of its own bacteriocin [57,58]. As above-indicated, LLBs are thought to be active immediately after their synthesis in the bacterial cytoplasm. Therefore, the self-immunity system would protect the producing strain from both intra- and extracellular molecules. Their intracellular toxicity was reported for EntDD14 and lacticin Q [47,52], but many questions related to this complex mechanism of immunity to LLBs remain to be answered. Recently, the analysis of the mutant deficient in the structural genes of EntDD14 demonstrated the implication of the precursor peptide in the self-immunity of the producer strain *E. faecalis* 14 [51]. In the case of aureocin 70A, the role of AurI in immunity was established and its heterologous expression in strains of Staphylococcus sensitive to aureocin 70 enabled them to become resistant to aureocin 70 [50]. Interestingly, the full immunity phenotype against aureocin 70 appeared to be dependent on the co-transcription of aurI and the putative transcriptional regulator orfA [50]. The best-known immunity system reported for LLBs is the ABC-type multi-drug resistance transporter protein. This ABC transporter was reported for many bacteriocins, including LLB (Figure 5), to be cyclic and non-leaderless [15,22,40,53,59]. This transporter was thought to play a role in both transport and immunity. It contributes to bacteriocin immunity by active extrusion of the peptide, most likely by reducing the concentration of the bacteriocin in direct contact with the cytoplasmic membrane [60,61,62]. These bacterial ABC transporters are composed of an ATP-binding protein and often one or more accessory proteins [63]. The deficiency of one more component of this ABC transporter affects the self-immunity system of the producing strains [21,53,64]. The transport of bacteriocins is most often mediated by an ABC transporter system [65] or in some cases by a secretory pathway [65]. As an example, the role of an ABC-type transporter AurT in the externalization of the aureocin A70A was reported [39]. Recently, the involvement of pleckstrin-homology-domain (PHD)-containing proteins in the transport of EntDD14 was established [52,53]. In light of this, the disruption of *orf8* encoding for PHD-containing proteins has abolished the production of aureocin A53 in *S. aureus* [47]. Interestingly, these PHD-containing proteins are found in many LLB as shown in Figure 5.

## 4. The Genetic and Synthesis Backgrounds of Enterocin DD14

The EntDD14 cluster encompasses 10 ORFs, namely *ddABCDEFGHIJ* [51]. Most of the genes present in the EntDD14 cluster are adjacent or overlap and in silico analysis suggested the presence of at least two operons. Thus, two putative transcriptional promoters and two putative terminators were detected. The first operon comprises *ddA* and *ddB* genes encoding the two-peptide LLB (DdA and DdB). Genes *ddA* and *ddB* are co-transcribed and their transcription is under the control of the putative P1 promoter, which is located upstream of the *ddA* gene (the P1 promoter presented a −35 (TTGAtA) and −10 (aATAAT) regions). The second contains the remaining genes *ddCDEFGHIJ* which encode two unknown proteins (DdC, D), membrane proteins YdbS and YdbT containing PHb2 domain DdE, F, and the ABC transporter (DdG, H, I and J) [51]. The transcription of this second operon seems to be dependent on the putative P2 promoter located upstream of the *ddC* gene (the P2 promoter presented a −35 (TTGttA) and a −10 (TATAtT) regions). The two putative ρ-independent transcriptional terminators designated T1 and T2 were identified between the 3’-end of *ddB* and the P2 promoter for T1 and the T2 at 163 nt downstream of the 3-end of the *ddJ* gene. 

**Figure 6:**
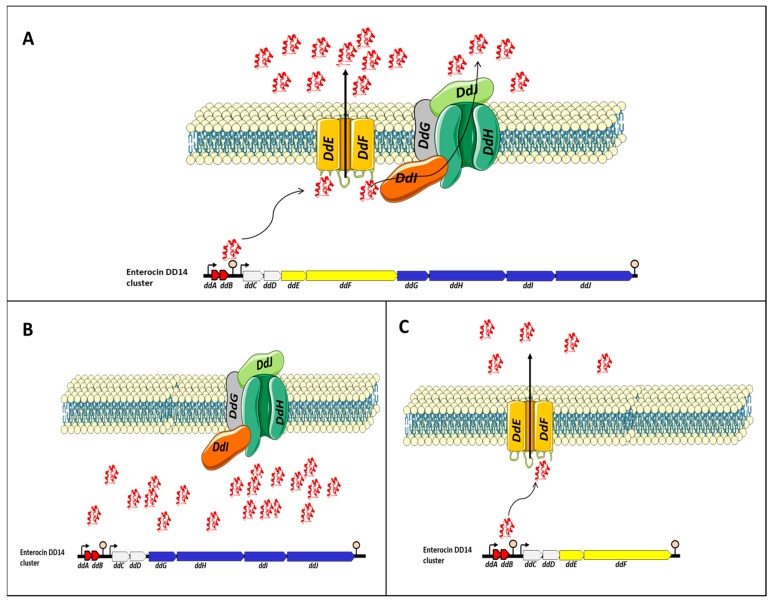
Proposed model for the transport of EntDD14. The legend is presented in the main text. (**A**) EntDD14 transport in the wild-type strain. (**B**) In the absence of DdE and/or DdF proteins, the strain was unable to export EntDD14 outside of the cytoplasm and an intracellular accumulation of EntDD14 was observed. (**C**) In the absence of one or more components the ABC transporter system (DdGHIJ), the bacteriocinogenic strain was still able to produce EntDD14, but at a lower rate than the wild-type.

To gain insights into the transport of EntDD14 and immunity mechanisms of *E. faecalis* 14, different derivative strains were constructed and their phenotype has been examined. The data gathered enabled us to suggest the model depicted in Figure 6. Transport of EntDD14 outside the cell is essentially ensured by a channel formed by DdE and DdF proteins [53]. This channel is used as a primary non-ATP-dependent transport to externalize EntDD14, until a certain threshold of extracellular bacteriocin concentration (Figure 6A), the bacteriocinogenic strain switches to the ABC transporter composed of DdGHIJ, which is an ATP-dependent transport. This ABC system requires ATP to transport the bacteriocin in the opposite of the concentration gradient. These two transport systems, namely DdEF and DdGHIJ, and ABC transporters (DdGHIJ) should interact synergistically to control the flow of bacteriocin, through a mechanism that needs to be studied. When DdE and/or DdF proteins are not produced (Figure 6B), the bacteriocinogenic strain was unable to export EntDD14 outside of the cytoplasm, despite the expression of genes coding for the ABC transporters and those coding for active EntDD14 (*ddAB*). The intracellular accumulation of EntDD14 was to be toxic for the bacteriocinogenic strain as a loss of 1 log viability was registered in a mutant deficient in *ddEF* [52]. When the ABC transporter system (DdGHIJ) was altered by deleting one or more components (Figure 6B), the bacteriocinogenic strain was still able to produce EntDD14, but at a lower rate than the wild-type [51,53]. These data indicate the role of ABC transporter in the transport of EntDD14, but not as the essential transporter. Nonetheless, we have also established its involvement in the protection of the bacteriocinogenic strain against extracellular EntDD14 [53].

## 5. Purification of Enterocin DD14

Despite their renewed interest in many applications, the purification of bacteriocins remains important in many cases, peculiarly in view of obtaining large amounts of purified and relatively clean active fraction which is generally difficult and costly [66,67]. The purification of bacteriocins, including leaderless ones, is usually carried out with a multistep procedure including ammonium sulfate precipitation, followed by one or more chromatography-based methods [66,67]. This strategy was largely utilized to purify bacteriocins devoid of leader peptide [38,68], such as enterocins L50A and L50B [20], A5-11A and A5-11B [37], and 62-6 [69]. Nonetheless, this strategy is time-consuming, expensive, and leads to low yields of purified peptides [67,70]. The ammonium sulfate precipitation enables a reduction in the volume of the culture supernatant and concomitantly increases the specific activity of the bacteriocin [71,72]. This step is usually not suitable for the purification of low molecular weight peptides due to the low precipitation yields and high saturation percentages [73,74,75], and this step requires a dialysis desalting phase, which can trigger significant loss of bacteriocins, because of their hydrophobic nature [75]. To overcome this drawback, easier methods of purification of bacteriocin were developed consisting of two-step-based methods. Such a simplified procedure was successfully applied for the purification of EntDD14 [33]. Accordingly, EntDD14 was purified using ion exchange chromatography and reverse phase HPLC, successively. To sum up, the producing strain, *E. faecalis* 14, was grown at 37 °C for 24 h with shaking (160 rpm), in an M17 medium supplemented with 5% glucose and buffered with 60 mM sodium phosphate (pH 6.3). After this period of incubation, the culture was centrifuged and the cell-free supernatant was incubated for 24 h at room temperature with CM Sephadex^®^ C-25 resin, which was resuspended and equilibrated in distilled water. The resin was then washed with distilled water and 0.5 M NaCl. Thus, EntDD14 bound to the resin was eluted with 1.5 M NaCl. Removal of the salt from the solution containing EntDD14 was performed by running on PD MidiTrap G-10 columns. This procedure is routinely performed and usually leads to better purification yields. An additional drying step using a speedvac-type rotary concentrator could be applied with the objective to store this peptide at 4 °C until its use [33,76]. To characterize the molecular mass of EntDD14A and EntDD14B, an additional separative step by the HPLC method is required, due to their close masses which are, respectively, 5200.74 Da and 5206.41 Da [33]. These molecular masses are comparable to those reported for L50A and L50B [20], MR10A and MR10B [34], 7A and 7B [35] and A5-11A and A5-11B [37]. It should be, however, mentioned that the purification of bacteriocins could be achieved using other strategies such as membrane separation, which has emerged and applied for the purification of diverse bacteriocins [77,78,79], including some enterocins [80,81].

## 6. Antibacterial Spectrum of Enterocin DD14 and Potentiation of Antibiotic Activity

Leaderless bacteriocins, mainly enterocins, are active against a wide range of Gram-positive pathogenic bacteria including *Listeria monocytogenes*, *Clostridium perfringens*, *Enterococcus faecalis* and *Staphylococcus aureus* [18,20,33,34,82]. Related to its spectrum of activity, EntDD14 was proven to be active against MRSA strains in both in vitro [83] and in vivo [84]. The combination of antimicrobials is foreseen to be a valuable tool allowing for the alleviation of bacterial resistance [85]. Related to this, we first reported the capability of nisin to potentiate the activity of colistin against Gram-negative bacilli [86]. Next, we established the abilities of EntDD28 and EntDD93, two bacteriocins closely related to EntDD14, to augment the activity of erythromycin or kanamycin against the MRSA-S1 strain [82], and that of EntDD14 to potentiate the activity of methicillin against different MRSA strains [83]. Remarkably, MRSA-S1 was resistant to erythromycin and kanamycin; however, if any of these antibiotics was used in combination with EntDD14, EntDD28, or EntDD93, the MRSA-S1 target strain returned to the sensitive phenotype. In addition, an anti-biofilm activity of EntDD28 and EntDD93 was demonstrated on the human MRSA strains [82]. In another study, we established that EntDD14 loaded on alginate nanoparticles has a more potent activity across MRSA strains [83], or *Clostridium perfringens* [76]. It is of note that treatment of *C. perfringens* with such nanobiotics (bacteriocins loaded on alginate nanoparticles) has annihilated the expression of genes encoding some virulence factors [76]. Recently, a study carried out in our laboratory has established an anti-adhesive action of EntDD14 against several MRSA strains, noticeably across *S. aureus* USA300, which is known for its virulence traits and ability to colonize different surfaces [87]. In this study, we also unveiled the potential of EntDD14 to decrease the secretion of pro-inflammatory interleukins, IL-6 and IL-8, on human intestinal Caco-2 cells previously inflamed with bacterial lipopolysaccharide [87]. Generally active only against closely phylogenetically related microorganism, some LAB-bacteriocins, however, are exceptionally active against Gram-negative bacilli [88,89]. EntDD14 is devoid of such antibacterial activity. Nevertheless, when EntDD14 was used in combination with colistin and tested against a panel of Gram-negative bacilli such as *Escherichia coli* strains, we could observe a better spectrum of colistin, arguing for a potentialization of this antibiotic by EntDD14 [90].

## 7. The Antiviral Spectrum of Enterocin DD14

Bacteriocins have already been described for their antiviral activity [87,91], and these include EntCRL35 or GEn17 on Herpes Simplex Virus-1 (HSV-1) [92,93]. Moreover, a study based on a global simulation approach established that bacteriocins warrant more attention for their potential against SARS-CoV-2 viruses [94]. Experimental data reported that some bacteriocins such as plantaricin or nisin were able to interact with enveloped viruses and spike protein, respectively [95,96]. It has been observed that EntDD14 has an antiviral activity in vitro against enveloped viruses (personal communication).

## 8. Cytotoxicity of Enterocin DD14

Bacteriocins such as nisin are used in the food industry as a food preservative and have been extensively studied for their cytotoxic effects on different cell lines. For example, nisin has been shown to have an IC50 of less than 10 µg·mL^−1^ in intestinal Caco-2 cells after 24 h of contact, and an IC50 of around 150 µg·mL^−1^ in Vero SF cells (monkey epithelial cells from kidney) [97]. In comparison, enterocin AS-48 (produced by *Enterococcus faecalis* UGRA 10) showed no cytotoxicity up to 200 µg·mL^−1^ on melanoma cell line A2058 [98]. In this context, EntDD14 was studied for its cytotoxic effects on several cell lines. First, the cytotoxicity of the purified enterocin DD14 was assessed using the IPEC-1 porcine intestinal epithelial cell line. After 24 h of contact, a dose-dependent decrease in IPEC-1 cell viability was observed when exposed to EntDD14. At concentrations of 50 μg·mL^−1^ and 100 μg·mL^−1^, reductions of 9.6% and 20% in cell viability were noted, respectively [32]. The cytotoxic effect of EntDD14 on two cell lines of human origin, intestinal Caco-2 cells, and HT29 mucus-producing cells was studied [76]. Thus, no apparent cytotoxicity was observed when EntDD14 was in contact with HT29 cells for 4 h and 24 h incubation and the two concentrations were tested (60 and 120 µg·mL^−1^). Regarding Caco-2 cells, the viability remained approximately 95% when exposed to a concentration of 120 µg·mL^−1^ of EntDD14 for 24 h [76]. Finally, another study (data under publication elsewhere) has shown the cytotoxic effects of EntDD14 on two cell lines, VERO E6 cells (derived from the kidney of an African green monkey) and Huh 7 cells (human hepatocellular carcinoma). For both cell lines, the viability decreased to 75% after 24 h incubation with EntDD14 at 120 µg·mL^−1^ (Submitted). To conclude, EntDD14 was found to be less cytotoxic than nisin.

## 9. Conclusions

**Figure 7:**
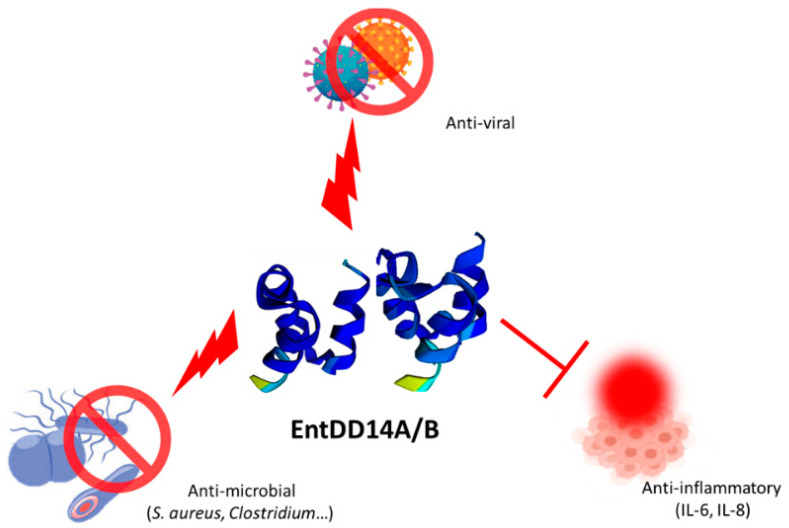
Multifunction activities of EntDD14 as anti-microbial against pathogenic Gram-positive bacteria, enveloped viruses and endowed with anti-inflammatory effects on IL-6 and IL-8 interleukins secretion in inflamed Caco-2 cells in-vitro.

LLBs are a very diverse group of bacteriocins endowed with common genetic, regulatory, and structural features. These bacteriocins were primarily known for the antimicrobial activities for which they were characterized. Among the LLBs, the two-peptide bacteriocins constitute a group of bacteriocins sharing very high similarities in their amino acid sequences and structures. EntDD14 was extensively studied as a model of two-peptide LLBs. The genetic determinants of EntDD14 as well as their organization into two distinct operons, namely *ddAB* and *ddCEFGHIJ*, were described. Next, the transport of EntDD14 was shown to be synergistically carried out by a central channel constituted by DdE/DdF pleckstrin-homology-domain carrying proteins, and the ABC transporter DdGHIJ. Furthermore, the immunity of the bacteriocinogenic *E. faecalis 14* towards extracellular EntDD14 was also discussed. In terms of spectrum, we noticed antibacterial activity across Gram-positive bacteria with in vitro and in vivo pieces of evidence. EntDD14 has also the ability to alleviate the synthesis of pro-inflammatory interleukins IL-6 and IL-8, delineating the multifunction activities of this LLB (Figure 7).

## Data Availability

All data are included within the manuscript.
